# Detection of epistasis interaction loci for fiber quality-related trait via 3VmrMLM in upland cotton

**DOI:** 10.3389/fpls.2023.1250161

**Published:** 2023-09-28

**Authors:** Zhimin Han, Huifeng Ke, Xiaoyu Li, Ruoxuan Peng, Dongdong Zhai, Yang Xu, Liqiang Wu, Wensheng Wang, Yanru Cui

**Affiliations:** ^1^ State Key Laboratory of North China Crop Improvement and Regulation, Key Laboratory for Crop Germplasm Resources of Hebei, Hebei Agricultural University, Baoding, China; ^2^ Jiangsu Key Laboratory of Crop Genomics and Molecular Breeding/Jiangsu Co-Innovation Center for Modern Production Technology of Grain Crops, College of Agriculture, Yangzhou University, Yangzhou, Jiangsu, China; ^3^ State Key Laboratory of Crop Gene Resources and Breeding, Institute of Crop Sciences, Chinese Academy of Agricultural Sciences, Beijing, China; ^4^ National Nanfan Research Institute (Sanya), Chinese Academy of Agricultural Sciences, Sanya, China

**Keywords:** cotton, fiber quality-related trait, GWAS, 3VmrMLM, epistasis interaction

## Abstract

Cotton fiber quality-related traits, such as fiber length, fiber strength, and fiber elongation, are affected by complex mechanisms controlled by multiple genes. Determining the QTN-by-QTN interactions (QQIs) associated with fiber quality-related traits is therefore essential for accelerating the genetic enhancement of cotton breeding. In this study, a natural population of 1,245 upland cotton varieties with 1,122,352 SNPs was used for detecting the main-effect QTNs and QQIs using the 3V multi-locus random-SNP-effect mixed linear model (3VmrMLM) method. A total of 171 significant main-effect QTNs and 42 QQIs were detected, of which 22 were both main-effect QTNs and QQIs. Of the detected 42 QQIs, a total of 13 significant loci and 5 candidate genes were reported in previous studies. Among the three interaction types, the AD interaction type has a preference for the trait of FE. Additionally, the QQIs have a substantial impact on the enhancement predictability for fiber quality-related traits. The study of QQIs is crucial for elucidating the genetic mechanism of cotton fiber quality and enhancing breeding efficiency.

## 1 Introduction

Cotton (*Gossypium* spp.) is one of the most important cash crops in the world, providing a large amount of natural fiber (Wendel and Cronn, 2003; Grover et al., 2014; Wang et al., 2021). Upland cotton (*Gossypium hirsutum* L.) was domesticated in the tropics under conditions high temperatures, plentiful rainfall and short days (He et al., 2021), accounting for approximately 95% of cotton production worldwide (Kumar et al., 2018). The fiber quality-related traits, such as fiber length (FL), fiber strength (FS), fiber elongation (FE) and fiber micronaire value (FM), which are closely related to the process of fiber cell development and differentiation including fiber initial differentiation stage, fiber elongation stage, secondary wall thickening stage and maturation stage (Pang et al., 2010). Therefore, the cotton fiber quality-related trait is a complex process involving co-expression and regulation of multiple genes, and consequently entails gene interactions.

Previous studies indicated that epistatic interactions account for a substantial proportion of the genetic basis of traits controlled by multiple genes (Liao et al., 2001). Epistasis refers to the interaction between alleles from different loci, which is the driving factor for the rapid evolution of traits and phenotypic diversity (Jia et al., 2014; Alonge et al., 2020; Liu et al., 2020a). In addition to main-effect quantitative trait nucleotides (QTNs), QTN-by-QTN interactions (QQIs) also play a significant role in gene expression and genetic variation. Many crops, such as rice (Li et al., 2001; Mei et al., 2005), Arabidopsis (Juenger et al., 2005), maize (McMullen et al., 2001), etc., have reported the significance of epistatic interactions as the genetic foundation of complex traits in recent years. In cotton quantitative trait genetic research, epistatic interactions have also been identified (Shen et al., 2006; Wang et al., 2006). Lin et al. (2009) performed main-effect QTNs and epistatic interaction QTNs analyses using a genetic linkage map of the F_2:3_ population containing 471 markers covering 65.88% of the whole cotton genome. There were a total of 9 main-effect QTNs associated with yield, 5 main-effect QTNs related to fiber quality traits, and 75 pairs of QQIs. This indicated that epistatic interaction effects played an important role in the inheritance of yield and fiber quality traits in upland cotton. Saha et al. (2011) performed partial diallel crosses with six chromosome substitution lines (CS-B lines), revealing additive, dominance and epistatic interaction effects for all fiber quality traits associated with CS-B lines. This indicated that epistatic interactions between the genes on the different chromosomes played a major role in the majority of the fiber quality traits. Wang et al. (2022) identified QTNs associated with fiber quality traits using a multi-parent advanced generation inter-cross (MAGIC) population of cotton and found that epistasis was universal. A total of 581 pairs of significant QQIs were identified for fiber-related traits, with the majority of epistatic pairs exhibiting moderate effects, explaining an average of 4% of the phenotypic variations. This indicated that epistasis played a crucial role in the variation of fiber quality-related traits. Despite the fact that epistasis interaction analyses have been reported for cotton, most of them concentrate on the genetic population rather than the natural population due to statistical method limitations.

Genome-wide association study (GWAS) has been widely used as an effective tool to detect QTNs related to target traits in many plants, such as wheat, rice, soybean, corn, cotton, etc (Chen et al., 2014; Juliana et al., 2019; Lu et al., 2020; Wang et al., 2020a). However, the majority of models only identify the main-effect QTNs, referred to as single-locus GWAS, so epistasis interaction QTNs were reported less frequently in GWAS. Fang et al (2017a); Fang et al., 2017b) used 318 local and modern improved varieties of cotton to detect 45 QTNs associated with fiber quality-related trait. Two loci associated with the ethylene pathway were found to be associated with fiber yield in their research. Wang et al. (2017) identified 19 candidate gene loci for fiber quality traits in 352 wild and domesticated cotton germplasms, of which 16 were newly identified QTNs. Ma et al. (2018) used 419 upland cotton core germplasms to detect 533 significant QTNs related to FS, one of which was validated for its functions. Although the majority of main-effect QTNs related to fiber quality have been reported in cotton, only a limited amount of phenotypic variation has been explained for fiber quality-related traits in cotton. Dissecting the epistasis interaction QTNs will contribute to a deeper understanding of the genetic mechanism underlying fiber quality-related traits controlled by multiple genes

In this study, we used a new R package called 3VmrMLM (Li et al., 2022a) developed by the team of Professor Yuanmin Zhang at Huazhong Agricultural University to detect the main-effect QTNs and QQIs in the upland cotton natural population including 1245 lines. The application of significant QQIs in cotton breeding and phenotypic prediction was discussed. The identification of favorable loci associated with cotton fiber quality will expedite the process of enhancing cotton fiber quality and provide a theoretical foundation for the genetic mechanism underlying cotton fiber quality.

## 2 Materials and methods

### 2.1 Plant materials

This study used a natural population including 1260 varieties of upland cotton from 3K-TCG panel including 3,278 accessions with 6,711,614 SNPs (He et al., 2021). Due to the limitation of population size, the materials were equally divided into two parts (*n*=630) and grown in four environments representing the major cotton cultivation regions, Yellow River region (YER), Yangtze River region (YZR), northern Xinjiang region and southern Xinjiang region, respectively. Each environment contains two locations and two replicates. YER is represented by the cities of Shijiazhuang in Hebei Province (38.22°N, 114.32°E) and Anyang in Henan Province (36.07°N, 114.50°E). YZR was represented by the cities of Yancheng in Jiangsu Province (33.34°N, 120.46°E) and Changsha in Hunan Province (28.38°N, 113.42°E). Two adjacent fields in Shihezi, Xinjiang Province (44.40°N, 86.16°E and 44.41°N, 86.71°E) represented the region of northern Xinjiang. South Xinjiang was represented by Kuche (41.82°N, 83.22°E) and Alaer (40.61°N, 81.33°E). Each line was grown with two random blocks and each block contained ~30 (YER and YZR) and ~60 (Xinjiang region) individuals. The field management is in strictly accordance with local planting standards.

In each block, cotton bolls with uniform development conditions were harvested for testing the fiber quality with a high-volume instrument (HVI9000) at the Urumqi Center of Supervision and Testing of Cotton Quality, Chinese Ministry of Agriculture. Fiber length (mm), fiber strength (cN/tex), and fiber elongation rate (%) were utilized to estimate the best linear unbiased prediction values (two replicates for two years) using the lme4 module of the R programming language (Bates et al., 2015). After discarding the accessions with low-quality data (missing or abnormal), a total of 1245 individuals with 1,122,352 SNPs (MAF > 0.05 and missing rate < 0.2) were retained for further GWAS analysis.

### 2.2 Introduction of 3VmrMLM

The 3VmrMLM method is a three-variance component mixed model method to detect and estimate all types of effects in GWAS. Our common genome-wide association analysis method is based on population structure and multi-gene background control and SNP fixed effect of single-marker association whole-genome scanning, which involves multiple tests. The 3VmrMLM method overcomes this limitation and greatly reduces the computational burden. It can compress the mixed model of five variance components into a mixed model of three variance components in main-effect QTNs detection, and compress the mixed model of 15 variance components into a mixed model of three variance components in QQIs detection.

Before using the 3VmrMLM method, the R package 3VmrMLM and its corresponding R packages must be locally installed in the R program, and the phenotype file, genotype file, and population structure file need to be prepared. Noteworthy is the fact that genotype data must be organized in plink format. This method can manage the entire SNP marker set for main-effect QTNs and environmental interaction QTNs. Keeping only 5000 SNPs (interaction pairs) is preferable for interaction QTNs due to the calculation time. Therefore, how the 5000 SNPs are selected is crucial to the QQI results. Users have to alter particular parameters to accomplish their own detection objectives.

### 2.3 Detection of main-effect QTNs

In this study, we used the 3VmrMLM method combining R and C++tools (Li et al., 2022b) to detect the main-effect QTNs for three traits to determine the association between genotypes and phenotypes. The significant associated main-effect QTNs were detected based on LOD = 3 threshold value. TASSEL v5.0 software was utilized to format the genotype files (Bradbury et al., 2007). Principal component analysis (PCA) was conducted using the PLINK v1.9 software (Purcell et al., 2007) as a population structure effect.

### 2.4 Detection of QQIs

Due to the “epistasis” method in the 3VmrMLM package needs a long running time, if all SNPs are operated, the work burden will be greatly increased, and the laptop will not be able to run. In order to save computing time and avoid missing significant loci, this study firstly screened the number of SNPs, and then carried out parameter modification and mapping analysis. The detailed steps are mainly divided into the following three steps:

In the first step, SNPs with *P* < 0.01 were screened out from the intermediate results of main effect QTNs detection for each trait. A total of 5822, 5370 and 4269 SNPs were screened out for FL, FS and FE, respectively. PLINK v1.9 software was utilized to convert the genotype files containing 1,122,352 SNPs into Plink binary files, from which the genotypes of SNPs screened for each trait were extracted.

In the second step, using the R package 3VmrMLM to detect QQIs with the specified parameters (blgwas_t = -2.5, svpal = (0.1, 0.1), and LOD = 3). TBtools v1.09 software was used to create a Circos diagram based on the results of QQIs (Chen et al., 2020).

In the third step, R ggplot2 package (Villanueva and Chen, 2019) was used to generate genotypic box plots of main-effect QTNs for different traits. Two-tailed Student’s *t*-test (Livak and Schmittgen, 2001) was conducted to test the significance between different haplotypes. The epistatic interactions of the two QTNs were depicted using line graphs, and their significance was determined by two-way analysis of variance (ANOVA) with ‘aov’ function of R software. Using the Chi-square independence test to evaluate the significance between genotypes and subgroups.

### 2.5 The mining of candidate genes

In this study, *Gossypium hirsutum* (AD1) ‘TM-1’ genome CRI v1.0 (Yang et al., 2019) was used as the reference genome, and relevant candidate genes were predicted by CottonFGD (Zhu et al., 2017). Due to the unequal distribution of genotypic SNPs, we regarded the main-effect QTNs and epistatic QTNs detected within the 200kb regions to be the same loci (Su et al., 2020). Co-localization QTNs are those that contain or overlap with previously reported QTNs within 200kb.

### 2.6 Predictability analysis

The rrBLUP package (Endelman, 2011) in R software was used to estimate the predictability of each trait. The main-effect QTNs and the combination of main-effect QTNs and QQIs were used to estimate the predictability by using ten-fold cross-validation. The missing genotypes were replaced by the average genotypic values. The predictability was calculated from the Person’s correlation coefficient between observed values and predictive values.

## 3 Results

### 3.1 The results of main-effect QTNs

A total of 171 QTNs were detected by the 3VmrMLM method, of which 62 QTNs related to FL were distributed on 24 chromosomes accounting for 31.21% phenotypic variation, 69 QTNs related to FS were identified on 24 chromosomes accounting for 33.54% phenotypic variation, and 40 QTNs related to FE were found on 18 chromosomes accounting for 37.58% phenotypic variation, respectively (**Supplementary Table 1**). Among the 62 QTNs for FL, the chromosome A07 contained the most with 6 QTNs, followed by the chromosome D11 with 5 QTNs. The remaining QTNs were located on the other 22 chromosomes. The PVE was explained by these 62 QTNs, which ranged from 0.17% to 3.08%. For the trait FS, the majority of QTNs were detected on chromosome A11, while the remaining QTNs were distributed on the remaining 23 chromosomes. The detected 69 QTNs carried an average PVE of 0.49%, with the range from 0.21% to 1.72%. The PVE was explained by the detected 40 QTNs associated with FE ranged from 0.29% to 6.43%, with an average of 0.94%.

We found that the majority of the main-effect QTNs only contributed a negligible portion (between 0.1% and 1%) of the phenotypic variation. Six QTNs contributed to PVE larger than 1.5%, of which *qFL23* is located on chromosome A09 with a *P*-value of 6.29×10^-125^, explaining 3.08% PVE. One QTN named *qFS23* was identified on chromosome A08 with a *P*-value of 8.55×10^-49^, which explained 1.72% PVE. The remaining four QTNs, *qFE10*, *qFE11*, *qFE25*, and *qFE27*, were detected on chromosomes A05, A09, D01 and D04 with the *P*-value of 5.43×10^-19^, 1.05×10^-21^, 7.42×10^-33^, 5.96×10^-57^, and explained 1.97%, 2.24%, 3.83% and 6.43% PVE, respectively (**Supplementary Table 1**). Within the candidate gene regions of the 171 QTNs, a total of 1063 candidate genes were detected, including 445 for FL, 378 for FS, and 240 for FE (**Supplementary Table 1**).

### 3.2 The results of QQIs

**Figure 1:**
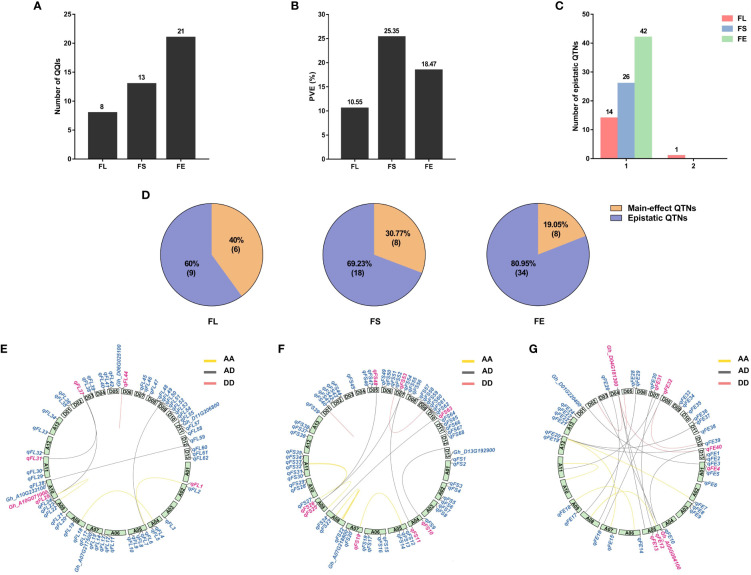
Characterization of QQIs contribution to phenotypic variance. FL, fiber length; FS, fiber strength; FE, fiber elongation. **(A)**: The number of QQIs for different traits. **(B)**: The percentage of phenotypic variance for different traits. **(C)**: Hotspots of epistatic interaction for different traits. 1 means one QTNs interacts with only one QTN. 2 means one QTN interacts with two QTNs. **(D)**: The proportion of main-effect QTN in QQI. Orange color, the number of main-effect QTNs in epistatic QTNs. Purple color, the number of epistatic QTNs excluding main-effect QTNs. **(E–G)**: Circos depicts the positions of the main-effect QTNs and their major candidate genes on different chromosomes. The internal line shows different epistasis interaction types. yellow line: AA types; red line: DD types; gray line: AD types; Blue labels outside circle: main-effect QTNs and the candidate genes reported previously. Pink labels outside circle, QTNs and candidate genes detected in both main-effect QTNs and epistatic QTNs.

A total of 42 pairs of QQIs were detected associated with the three fiber-quality traits, explaining 54.37% of the a cumulative PVE. Eight pairs of QQIs for FL, explaining 10.55% cumulative PVE, were identified with an average PVE of 1.32%, ranging from 0.27% to 5.69%. A total of 13 pairs of QQIs were detected in relation to FS, with PVE ranging from 0.23% to 5.55%, carrying an average PVE of 1.95% and cumulative PVE of 25.35%. For the trait FE, 21 pairs of QQIs were found, accounting for 18.47% of phenotypic variation, with an average PVE of 0.88% ranging from 0.36% to 2.17% (**Table 1**; **Figures 1A, B**). The results revealed that the cumulative PVE explained by the QQIs was not increased according to the increased number of QQIs detected in different traits.

**Table 1:** The QQIs for fiber quality-related traits.

QTN_1	Chromosome_1	Position_1 (bp)	QTN_2	Chromsome_2	Position_2 (bp)	LOD	PVE (%)	*P*-value
***epiFL-A02-1***	A02	161178	*epiFL-A05-1*	A05	1025103	4.26	1.11	5.96E-04
*epiFL-A04-1*	A04	48554477	*epiFL-A08-1*	A08	31754811	3.05	0.27	7.13E-03
*epiFL-A05-2*	A05	5682622	*epiFL-D09-1*	D09	5096223	3.9	0.63	1.26E-03
***epiFL-A09-1***	A09	77814014	***epiFL-A10-1***	A10	13480411	3.14	5.69	5.92E-03
***epiFL-A10-1***	A10	13480411	*epiFL-D03-1*	D03	378922	4.41	1.05	4.36E-04
*epiFL-A10-2*	A10	111356427	*epiFL-D12-1*	D12	58183426	3.69	0.51	1.92E-03
***epiFL-A11-1***	A11	115361620	***epiFL-D03-2***	D03	2555685	3.37	0.3	3.77E-03
***epiFL-D06-1***	D06	4166176	*epiFL-D06-2*	D06	4179009	3.52	0.99	2.73E-03
***epiFS-A04-1***	A04	6079915	*epiFS-D13-1*	D13	1935015	3.79	1.54	1.56E-03
***epiFS-A04-2***	A04	77171255	***epiFS-D07-3***	D07	42476906	5.77	2.56	2.41E-05
*epiFS-A05-1*	A05	22375825	***epiFS-A07-1***	A07	17653715	4.8	0.54	1.93E-04
*epiFS-A08-1*	A08	23106306	*epiFS-A08-3*	A08	31554661	4.79	5.55	1.97E-04
*epiFS-A08-2*	A08	25927381	*epiFS-A08-4*	A08	72822991	6.79	2.06	2.72E-06
*epiFS-A08-5*	A08	111030143	*epiFS-D10-1*	D10	62450484	3.32	1.88	4.16E-03
*epiFS-A09-1*	A09	1406341	***epiFS-A09-6***	A09	79606758	3.95	2.77	1.14E-03
*epiFS-A09-2*	A09	61235738	***epiFS-D05-1***	D05	52213369	4.46	0.23	3.89E-04
*epiFS-A09-3*	A09	61429015	*epiFS-A11-1*	A11	72500948	3.39	0.77	3.59E-03
***epiFS-A09-4***	A09	61531557	*epiFS-D07-2*	D07	41487983	3.97	3.78	1.08E-03
*epiFS-A09-5*	A09	61759908	*epiFS-A11-2*	A11	77324419	3.87	0.44	1.34E-03
*epiFS-D01-1*	D01	44446941	*epiFS-D01-2*	D01	44526524	4.98	0.86	1.32E-04
*epiFS-D07-1*	D07	2089762	***epiFS-D09-1***	D09	50463673	3.63	2.37	2.18E-03
***epiFE-A01-1***	A01	11746972	*epiFE-D02-1*	D02	911647	3.59	0.63	2.39E-03
*epiFE-A02-1*	A02	101002877	*epiFE-A12-3*	A12	102677250	5.32	0.4	6.37E-05
***epiFE-A05-1***	A05	9995639	*epiFE-D03-1*	D03	2892564	3.04	0.78	7.28E-03
*epiFE-A05-2*	A05	10502078	*epiFE-D13-2*	D13	5019567	3.09	0.7	6.64E-03
***epiFE-A05-3***	A05	23699241	*epiFE-D04-3*	D04	51418498	3.95	0.72	1.14E-03
*epiFE-A05-4*	A05	23704959	*epiFE-A08-2*	A08	68484251	5.34	1.07	6.04E-05
***epiFE-A05-5***	A05	51399299	***epiFE-D07-1***	D07	27619443	3.59	0.37	2.58E-04
*epiFE-A05-6*	A05	94562331	*epiFE-D04-5*	D04	51927145	4.77	0.72	2.05E-04
*epiFE-A06-1*	A06	34388517	*epiFE-A09-1*	A09	69963530	3.75	0.39	1.72E-03
*epiFE-A07-1*	A07	42472572	*epiFE-A12-1*	A12	98605178	4.43	0.79	4.20E-04
*epiFE-A07-2*	A07	89882612	*epiFE-D08-2*	D08	4292304	3.79	0.36	1.56E-03
*epiFE-A08-1*	A08	11134108	*epiFE-D04-1*	D04	8173930	6.15	0.54	1.08E-05
*epiFE-A08-3*	A08	113486209	*epiFE-D06-1*	D06	2210762	6.08	1.88	1.25E-05
*epiFE-A10-1*	A10	112466148	*epiFE-A11-2*	A11	84074705	4.16	0.55	7.34E-04
*epiFE-A11-1*	A11	47980891	*epiFE-D07-2*	D07	47702223	4.53	0.82	3.35E-04
*epiFE-A12-2*	A12	102606983	*epiFE-D12-2*	D12	61076721	3.73	1	1.78E-03
*epiFE-A12-4*	A12	104448140	*epiFE-D11-1*	D11	24596658	3.93	2.16	1.19E-03
*epiFE-D02-2*	D02	69758057	*epiFE-D04-2*	D04	51351966	3.28	0.68	4.46E-03
*epiFE-D04-4*	D04	51635984	*epiFE-D05-1*	D05	10091712	3.98	0.87	1.07E-03
***epiFE-D04-6***	D04	52433913	***epiFE-D13-1***	D13	4805762	5.66	2.17	3.09E-05
***epiFE-D08-1***	D08	3966472	*epiFE-D12-1*	D12	44788680	3.13	0.89	6.03E-03

QTN is named as follows: epi + traits abbreviation + chromosome + QTN number.

Bold indicates the loci detected in both main-effect QTNs and QQIs detection.

In comparison to the results of main-effect QTNs, we categorized the QQIs into three types. Type I was defined as the detected pairs of QQIs that were also identified as the main-effect QTNs. Type II was defined as one of the QQIs detectable as the main-effect QTNs. Type III was defined as none of QQIs can be identified as the main-effect QTNs. The proportion of type I QQIs was very low, only two pairs of QQIs were found in FL (*epiFL-A09-1* by *epiFL-A10-1*, *epiFL-A11-1* by *epiFL-D03-2*) with the PVE of 5.69% and 0.30%, respectively; One pair of QQIs was found in FS (*epiFS-A04-2* by *epiFS-D07-3*), with the PVE of 2.56%; Two pairs of QQIs were found in FE (*epiFE-A05-5* by *epiFE-D07-1*, *epiFE-D04-6* by *epiFE-D13-1*) with the PVE of 0.37% and 2.17%, respectively. A total of 13 pairs of type II QQIs were identified, including 3 pairs for FL, 6 pairs for FS, and 4 pairs for FE, with the cumulative PVE of 3.15%, 11.24% and 3.02%, respectively. The proportion of type III QQIs was higher than the other types, a total of 24 pairs of QQIs were detected, including 3 pairs for FL, 6 pairs for FS, and 15 pairs for FE, with the cumulative PVE of 1.41%, 11.56% and 12.91%, respectively (**Table 1**). According to the above results, we found that 22 loci were both main-effect QTNs and epistasis QTNs for the three fiber-quality traits. The percentages were as follows: 40% for FL, 30.77% for FS, and 19.05% for FE (**Figure 1D**). The overlapping ratio between main-effect and epistasis is significantly lower for FE than that of the other two traits. This indicated that epistasis interaction, particularly the type III epistasis, has a substantial effect on FE.

The epistasis interaction based on the subgenome was also examined. There were three types: AA (loci interactions between At subgenome), DD (loci interactions between Dt subgenome) and AD (loci interactions between At subgenome and Dt subgenome). Among the 42 pairs of QQIs, 14 pairs belonged to the AA types, explaining 22.41% of the cumulative phenotypic variation, with an average PVE of 1.60% that varied from 0.27% to 5.69%. The proportion of DD types was the lowest, only 7 pairs of QQIs were detected with the PVE ranged from 0.68% to 2.37%, explaining 1.26% of the phenotypic variation on average and 8.82% of cumulative phenotypic variation. A total of 21 pairs of QQIs were AD types, explaining 23.14% of the cumulative phenotypic variation, with an average PVE of 1.10% ranging from 0.23% to 3.78% (**Supplementary Table 2**; **Figures 1E–G**).

For the trait of FL, the AD type interaction was the most common, including four pairs of QQIs, accounting for 2.49% of the cumulative phenotypic variation and 0.62% of the average PVE. The DD type interaction was the least, only one pair of QQI was detected with an average PVE of 0.99%. There were three pairs of AA types explaining 2.36% of the average PVE and 7.08% of the cumulative PVE (**Figures 1E**; **Supplementary Table 2**). For the trait FS, there were six and five pairs of AA and AD interaction types, with the PVE ranging from 0.44% to 5.55% and 0.23% to 3.78%, respectively. Their average PVE was 2.02% and 2.00% and the cumulative PVE was 12.14% and 9.98%. Only two pairs of DD types were detected with an average PVE of 1.61% and cumulative PVE of 3.23% (**Figure 1F**; **Supplementary Table 2**). For the trait of FE, there were five pairs of AA interactions with 0.64% of average PVE, explaining 3.20% of cumulative PVE. The number of AD and DD types was 12 and 4, with 0.89% and 1.15% of the average PVE and 10.67% and 4.60% of the cumulative PVE. The proportion of AD interaction was the highest for FE compared to the other traits (**Figures 1G**; **Supplementary Table 2**).

Among the detected QQIs, one epistatic hotspot was found in relation to FL which interacted with two QTNs (**Figure 1C**), whereas the remaining epistatic QTNs only interacted with one QTN. Results of main-effect QTNs and QQIs for the three traits were showed on **Figures 1E–G**. For FL, five main-effect QTNs (*qFL1*、*qFL25*、*qFL31*、*qFL37*、*qFL44*) and one candidate gene (*Gh_A10G071000*) were detected as the epistatic QTNs. There being 8 main-effect QTNs named *qFS10*、*qFS11*、*qFS19*、*qFS25*、*qFS26*、*qFS48*、*qFS53*、and *qFS63* were detected as epistatic QTNs in FS. For FE, six epistatic QTNs and two candidate genes were detected as main-effect QTNs.

### 3.3 Analysis for the important QQIs

The 1,245 lines were classified into five subgroups (G1-G5) according to the genotype information-based population structure analysis, phylogenetic analysis and principal component analysis (PCA) (He et al., 2021). One individual (GH0186) was not included in the five subgroups (**Supplementary Table 3**). The accessions collected in South China (SC) were classified into G1 (n=12) subgroup. The G2 (n=155) subgroup comprised the majority of early-maturity accessions, which were primarily cultivated in Northwest China (NWC) and North China (NC). The G3 (n=220) subgroup was composed of the accessions collected from all three historical Chinese cotton planting areas. The G4 (n=194) subgroup included 194 accessions, most of which were cultivated in the Yangtze River region (YZR) south of China. The last group G5 (n=663) has the greatest number of accessions, which mainly come from the Yellow River region (YER) and the United States. We illustrate the application of epistatic effects in cotton breeding with two examples, including the combination of two important QQIs with subgroup information.

**Figure 2:**
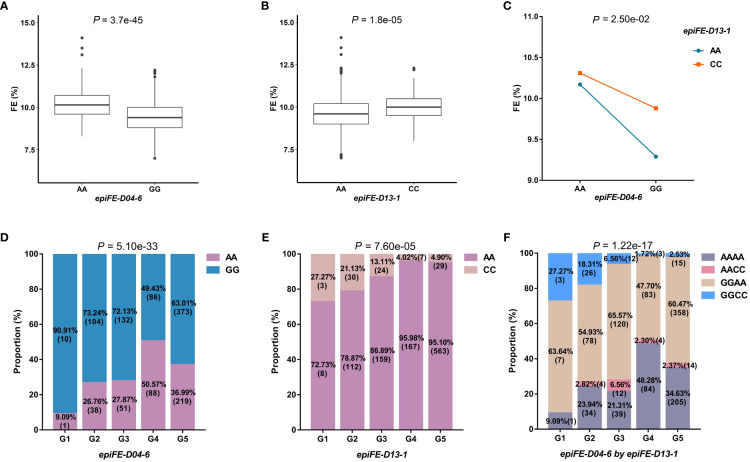
Characterization of important QQIs for FE. FE, fiber elongation. **(A)**: Boxplot of FE in two genotypes at *epiFE-D04-6*. The significance of difference between the two genotypes was analyzed with two tailed *t*-test. **(B)**: Boxplot of FE in two genotypes at *epiFE-D13-1*. **(C)**: Interaction plot for epistasis between two QTNs (*epiFE-D04-6* by *epiFE-D13-1*). The significance of the interaction between the two loci was analyzed with two-way analysis of variance. **(D)**: The proportion of two genotypes in different subgroups at *epiFE-D04-6*. **(E)**: The proportion of two genotypes in different subgroups at *epiFE-D13-1*. Chi-square independence test was used to analyze the significance of genotype difference among different subgroups. **(F)**: The proportion of different genotype combinations in different subgroups for QQIs.

The first example was *epiFE-D04-6*, interacting with *epiFE-D13-1*, which was located in the same region of the gene *Gh_D04G181300* reported previously in relation to FE (Thyssen et al., 2019). The two QTNs were also detected as the main-effect QTNs, which belong to the epistasis type I. The phenotypic values of the two genotypes (AA-GG and AA-CC) at the two loci of *epiFE-D04-6* and *epiFE-D13-1* were both extremely significant difference in the *t*-test (*P*-value < 0.01). Mean FE values of AA and GG were 10.19% and 9.36% at *epiFE-D04-6*, mean FE values of AA and CC were 9.61% and 10.03% at *epiFE-D13-1*, respectively (**Figures 2A, B**). The mean phenotypic value of the individual with superior allele (genotype AA) at the *epiFE-D04-6* was enhanced by the genotype of CC at *epiFE-D13-1*. Analysis of variance revealed a significantly distinct interaction effect (**Figure 2C**). Excluding the missing and heterozygous genotypes, the numbers of lines carrying the two QTNs belonged to the five subgroups were 11, 142, 183, 174 and 592, respectively. The chi-square test revealed a significant difference between the genotypes of the two QTNs and the five subgroups, indicating that subgroups influenced the genotypic selection. A total of 50.57% of individuals carried the superior allele of *epiFE-D04-6* (AA) in G4 subgroup, indicating that the superior allele was strongly selected in G4 subgroup (**Figure 2D**). Superior allele of *epiFE-D13-1* (CC) was strongly selected in G1 subgroup. A total of 27.27% of individuals carried superior allele of *epiFE-D13-1* (CC) in G1 subgroup (**Figure 2E**), which were mainly from southern China. The proportion of individuals carrying the AACC genotype was 6.56% in G3 subgroup, which is higher than the joint probability of genotype of AA in *epiFE-D04-6* and genotype of CC in *epiFE-D13-1* (3.65%). Therefore, superior allele of combination of *epiFE-D04-6* and *epiFE-D13-1* (AACC) was strongly selected in G3 subgroup (**Figure 2F**).

**Figure 3:**
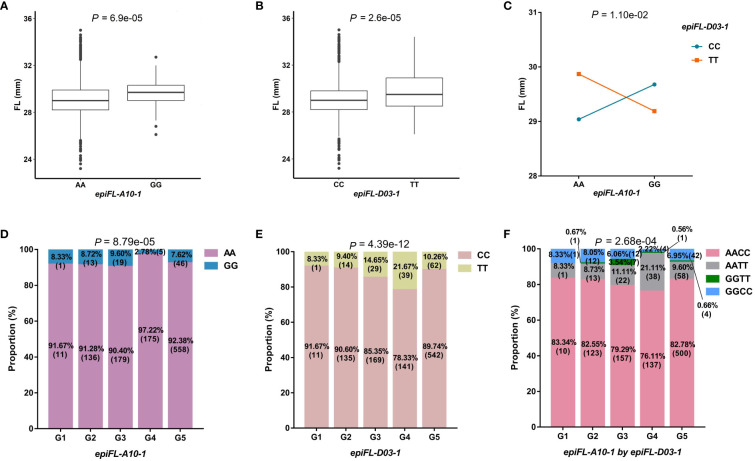
Characterization of important QQIs for FL. FL, fiber length. **(A)**: Boxplot of FL in two genotypes at *epiFL-A10-1*. The significance of difference between the two genotypes was analyzed with two tailed *t*-test. **(B)**: Boxplot of FL in two genotypes at *epiFL-D03-1*. **(C)**: Interaction plot for epistasis between two QTNs (*epiFL-A10-1* by *epiFL-D03-1*). The significance of the interaction between the two loci was analyzed with two-way analysis of variance. **(D)**: The proportion of two genotypes in different subgroups at *epiFL-A10-1*. Chi-square independence test was used to analyze the significance of genotype difference among different subgroups. **(E)**: The proportion of two genotypes in different subgroups at *epiFL-D03-1*. **(F)**: The proportion of different genotype combinations in different subgroups for QQIs.

The second example was the epistasis hotspot of *epiFL-A10-1* interacted with *epiFL-D03-1* for FL. The *epiFL-A10-1* was identified on the same region to the previous reported *Gh_A10G071000* (Geng et al., 2020). The difference between two genotypic values of *epiFL-A10-1*, 29.17mm for AA and 29.62mm for GG, reached a significant level (**Figure 3A**). There was also significant difference between the two genotypic values of *epiFL-D03-1* with CC for 29.09mm and TT for 29.81mm (**Figure 3B**). There was a very significant difference between the genotypic values of AACC and AATT. The mean phenotypic value of the plants with allele of *epiFL-A10-1* (genotype AA) was enhanced by the genotype of TT of *epiFL-D03-1* (the phenotypic values of AACC and AATT were 29.04mm and 29.87mm, respectively) (**Figure 3C**). Without considering missing and heterozygosis genotypes at the two loci, the population size of the five subgroups was 12, 149, 198, 180, and 604, respectively. The chi-square test showed that there were significant differences between genotypes of the two loci and subpopulations (**Figures 3D–F**), indicating that genotype was selected by subpopulation. Among the five subgroups, the proportion of individuals carrying allele of *epiFL-A10-1* in G4 was 97.22% which was higher than that in the other subgroups. Most of these individuals were from the Yangtze River Basin. The proportion of individuals carrying the favorable alleles of *epiFL-D03-1* in G4 subgroup was the highest, reaching 21.67% (**Supplementary Table 3**; **Figure 3E**). In the interaction between *epiFL-A10-1* and *epiFL-D03-1*, the combined genotype AATT accounted for the largest proportion in the G4 subgroup (**Figure 3F**). In addition, the joint ratio of AA genotype in *epiFL-A10-1* and dominant allele TT in *epiFL-D03-1* in G4 subgroup (21.07%) was lower than that in their interaction (21.11%), so the dominant genotype combination (AATT) was strongly selected in G4 subgroup.

### 3.4 The effect of QQIs for genomic prediction

**Figure 4:**
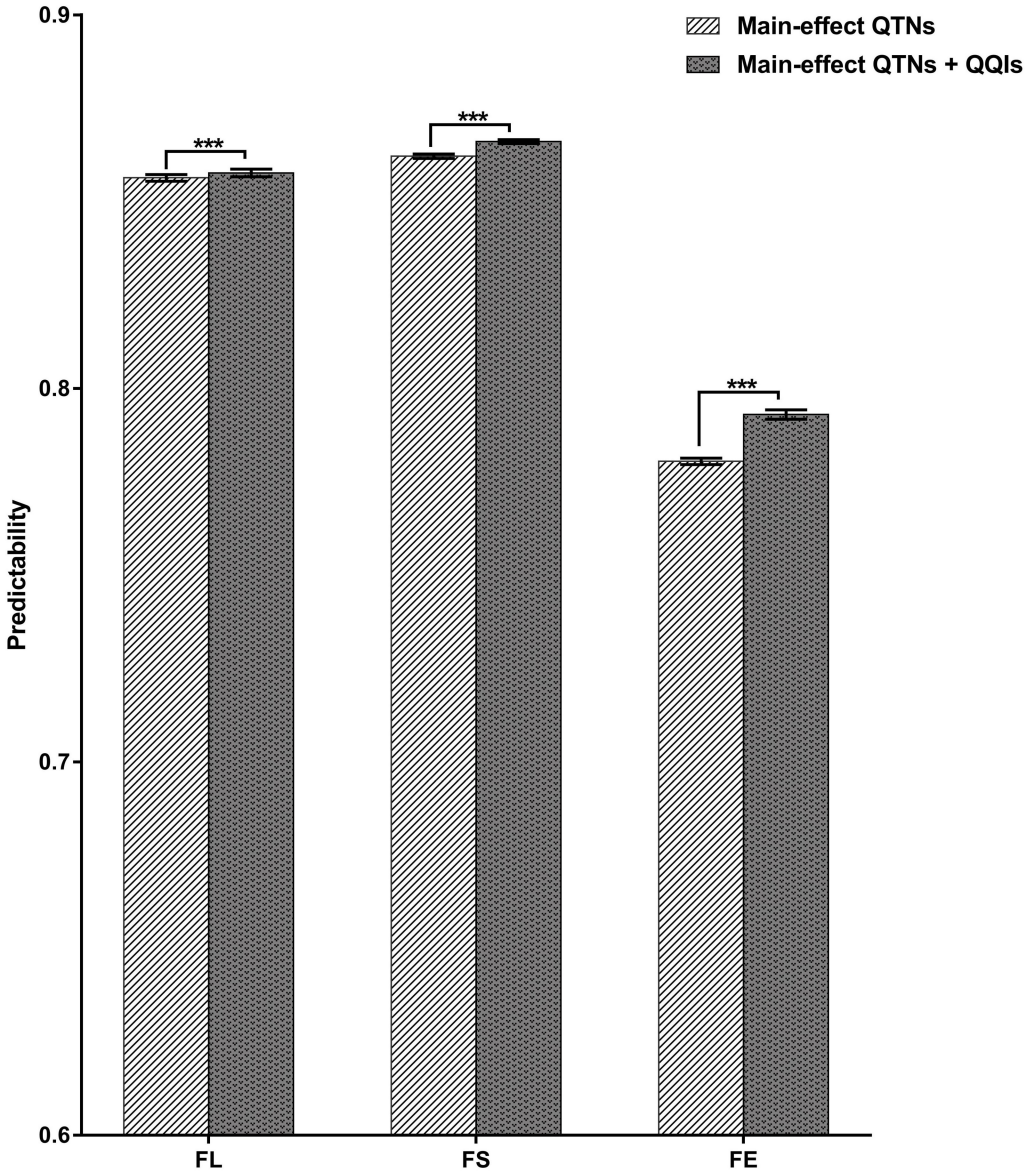
Predictabilities for the three traits using different QTNs. FL, fiber length; FS, fiber strength; FE, fiber elongation. Comparison of predictability (*R*) between two types QTNs for different traits based on the 10-fold cross validation method. *** indicates significant difference at *P* < 0.001 (two tailed *t*-test).

We compared predictability of each trait estimated from the main-effect QTNs, joint main-effect QTNs and QQIs through 10-fold cross validation (**Figure 4**). The predictability for the three traits (FL, FS, FE) were 0.8563, 0.8622 and 0.7805 estimated from main-effect QTNs, 0.8577, 0.8660 and 0.7930 estimated from the joint data, respectively. The results showed that the predictability of each trait estimated from joint data were all higher than that derived from the main-effect QTNs.

## 4 Discussion

### 4.1 The importance of QTNs and QQIs to dissect the genetic mechanism in cotton

GWAS has been widely used to identify the complex quantitative traits and dissect genetic mechanism in plants. However, most of the GWAS method focus on the high-density single SNP analysis considering the multiple tests. In this study, we performed multiple-locus GWAS method from 3VmrMLM to identify the main-effect and epistasis QTNs. The number of main-effect QTNs we detected using 3VmrMLM was higher than that was detected by professor Du’s team using EMMAX (Kang et al., 2010) method. The 3VmrMLM method is a compressed variance component mixed model to detect all types of loci and almost unbiasedly estimated their effects. In most existing methods and software of GWAS for detecting quantitative trait nucleotides (QTNs), QTN-by-environment interactions (QEIs), and QTN-by-QTN interactions (QQIs), only the allele substitution effect and its interaction-related effects are detected and estimated, conditional on method-specific polygenic background control, leading to confounding in effect estimation and insufficient polygenic background control. The 3VmrMLM overcomes the limitation to estimate additive and dominant effects and their environmental and epistatic interaction effects, conditional on fully controlling all possible polygenic backgrounds (Li et al., 2022a). Moreover, the 3VmrMLM method greatly reduces the computational burden. It can compress the mixed model of five variance components into a mixed model of three variance components in main-effect QTNs detection, and compress the mixed model of 15 variance components into a mixed model of three variance components in QQIs detection. In this study, a total of 171 fiber quality-related main-effect QTNs were detected, of which 10 genes were reported in previous studies and 21 QTNs located on the same candidate regions of the previous results (**Supplementary Table 1**) (Huang et al., 2017; Sun et al., 2017; Dong et al., 2018; Li et al., 2018; Ma et al., 2018; Thyssen et al., 2019; Geng et al., 2020; Li et al., 2020; Liu et al., 2020b; Nazir et al., 2020; He et al., 2021; Song et al., 2021; Wang et al., 2022). For FL, we detected the candidate gene *Gh_D11G206800*, which corresponds to the previously confirmed gene *Gh_D11G1929*, to be highly expressed during fiber development from 5 to 20 days post anthesis (DPA) (Sun et al., 2017; Ma et al., 2018). We further found that *Gh_D11G206800* is homologous to Arabidopsis *AT3G19150* gene, which encodes KIP-related protein 6 (KRP6) affecting the expression of cell wall organization to regulate cell elongation (Jegu et al., 2013; Li et al., 2020). The detected gene *Gh_A07G217500* is identical with the previously reported gene *Gh_A07G1758* (Sun et al., 2017) which is homologous to *AT4G17170* coding Ras-related protein RABB1c in Arabidopsis. Moreover, the results of transcriptome analysis showed that *Gh_A07G217500* exhibited dynamic changes in 5-20 DPA during the development of fiber cells, and was highly expressed at 10-15DPA, thereby accelerating the elongation of fiber cells (Sun et al., 2017). Therefore, *Gh_D11G206800* and *Gh_A07G217500* are candidate genes for FL. For FS, the identified gene *Gh_A07G218800* was consistent with previously reported gene *Gh_A07G1769*, which contains two haplotypes with significantly different fiber strength (Sun et al., 2017; Ma et al., 2018; Thyssen et al., 2019; Geng et al., 2020; Wang et al., 2022). Therefore, we infer that this gene is a causal gene affecting cotton fiber strength. The same region of the gene *Gh_D01G22040* was reported previously in relation to FE (Thyssen et al., 2019; He et al., 2021; Wang et al., 2022). Through the sequence alignment, we found that *Gh_D01G22040* gene is homologous to Arabidopsis *AT2G35880* gene, which is a member of the TPX2 gene family and plays a vital role in plant development by coding for the protein WVD2-like 4 to regulate the dynamic changes of the microtubule. Microtubule is essential for the fiber development, and thus this gene may be a candidate gene for FE.

Among the detected 42 QQIs, 12 epistasis QTNs were consistent with previous studies (**Supplementary Table 2**) (Huang et al., 2017; Sun et al., 2017; Wang et al., 2017; Li et al., 2018; Ma et al., 2018; Thyssen et al., 2019; Geng et al., 2020; Li et al., 2020; Nazir et al., 2020; He et al., 2021; Song et al., 2021). The detected QQIs have an important contribution to the improvement of cotton fiber quality. The superior genotype combination AACC was obtained from the interaction between *epiFE-D04-6* and *epiFE-D13-1*, carrying the highest FE. The gene *Gh_D04G181300* located on the candidate region of *epiFE-D04-6*, showing a dynamic change in 5-20DPA during the development of fiber cell. In addition, the transcriptome analysis showed that *Gh_D04G181300* was highly expressed in both ovule development and fiber cell development, thus affecting cotton fiber elongation (He et al., 2021). We inferred that interaction between *epiFE-D04-6* and *epiFE-D13-1* has a significant contribution to the improvement of FE in cotton, but its role in the molecular mechanism requires to be further verified. The superior genotype combination AATT was raised from the interaction between *epiFL-A10-1* and *epiFL-D03-1* for increasing the FL in cotton. The candidate region of *epiFL-A10-1* contains *Gh_A10G071000* which was previously reported in relation to FL of cotton, but its role in the molecular mechanism needs to be further verified (Geng et al., 2020).

The previous study showed that the expression of gene related to fiber development are inhibited in haploids containing only the D subgenome, which promotes the expression of gene related to fiber development when the A and D subgenomes are combined (Applequist et al., 2001). This viewpoint is well supported by the analysis of subgenomic interaction types in this study. In our study, the PVE estimated from AD interaction types was 14.32% higher than that from DD interaction types. Moreover, we found that AD interaction type was favorable for FE, which is consistent to the previous studies (Wang et al., 2022). The proportion of AD interaction type was 57.14% for FE which was higher than that for FL and FS. The AD interaction type explained 10.67% of PVE for FE, which was the largest compared with FL (2.49%) and FS (5.55%).

### 4.2 The application of epistasis QTNs in crop breeding

The QQIs substantially contribute to the explanation the phenotypic variation of quantitative traits (Carlborg and Haley, 2004; Wang et al., 2020b). In this study, the QQIs detected for fiber quality-related traits accounted for 54.37% of the phenotypic variation. For each trait, QQIs contributed the most to FS, accounting for 25.35% of the PVE, indicating that epistatic effects were the primary factor controlling the variation of FS. In the analysis of predictability for the three traits, the predictabilities were all improved by using the joint data (main-effect QTNs and epistasis QTNs) compared to only using main-effect QTNs, but the improvement was not substantial. One of the possible reasons is that only 5000 SNPs were used to detect epistasis QTNs at a time in the 3VmrMLM method. The 5000 SNPs were selected from the single locus GWAS based on a relatively low threshold, such as 0.05. Therefore, the significant epistasis QTNs were not detected on the scope of the entire genome, and it is likely that some potential epistasis QTNs that were not significant based on the single locus GWAS were missed. The development of a statistical method of epistatic interaction GWAS that takes into account the entire genome is a promising direction.

Individuals in the G3 subgroup were predominantly from Xinjiang with the largest average of 9.69% of FE (**Supplementary Figure 1A**). In addition, interaction analysis between *epiFE-D04-6* and *epiFE-D13-1* revealed that the superior genotype combination AACC was strongly selected in the G3 subgroup. Therefore, we infer that the high value of FE in the G3 subgroup was partly due to the contribution of the superior genotype combination AACC, and plants with high FE were readily obtained from upland cotton planted in this area. Materials of G4 subgroup were mainly from the Yangtze River Basin in Jiangsu, and its FL value (29.63 mm) was considerably higher than those of the other four subgroups (**Supplementary Figure 1B**). Moreover, through interaction analysis between *epiFL-A10-1* and *epiFL-D03-1*, we found that the superior genotype combination AATT was strongly selected in the G4 subgroup. Therefore, we speculated that the high FL in G4 subgroup was partly contributed by the superior genotype combination AATT. Growing upland cotton in this region is conducive to producing long FL plants. Future research can develop efficient molecular markers targeting these epistatic interaction QTNs to expedite the application of molecular marker-assisted selection in cotton breeding and enhance cotton breeding efficiency for fiber quality.

In conclusion, the detection of QQIs is extremely important for the investigation of the genetic mechanism underlying cotton fiber quality-related traits. This study will further accelerate the process of epistatic interaction in the study of cotton fiber quality, help to dissect the genetic mechanisms of cotton fiber quality, and promote the breeding of upland cotton plants with excellent fiber quality.

## Data availability statement

The original contributions presented in the study are included in the article/**Supplementary Material**. Further inquiries can be directed to the corresponding author.

## Author contributions

YC and LW designed the project and ZH analyzed the data and wrote the manuscript. HK, XL, RP, DZ and YX assisted in conducting data analysis. WW and YC provided the direction for the study and the correction of the manuscript. All authors contributed to the article and approved the submitted version.
