# Supplementary figures and images for: Detection of epistasis interaction loci for fiber quality-related trait via 3VmrMLM in upland cotton
Source: Front Plant Sci. 2023 Sep 28;14:1250161. doi: 10.3389/fpls.2023.1250161 (PMC10568130; doi:10.3389/fpls.2023.1250161)

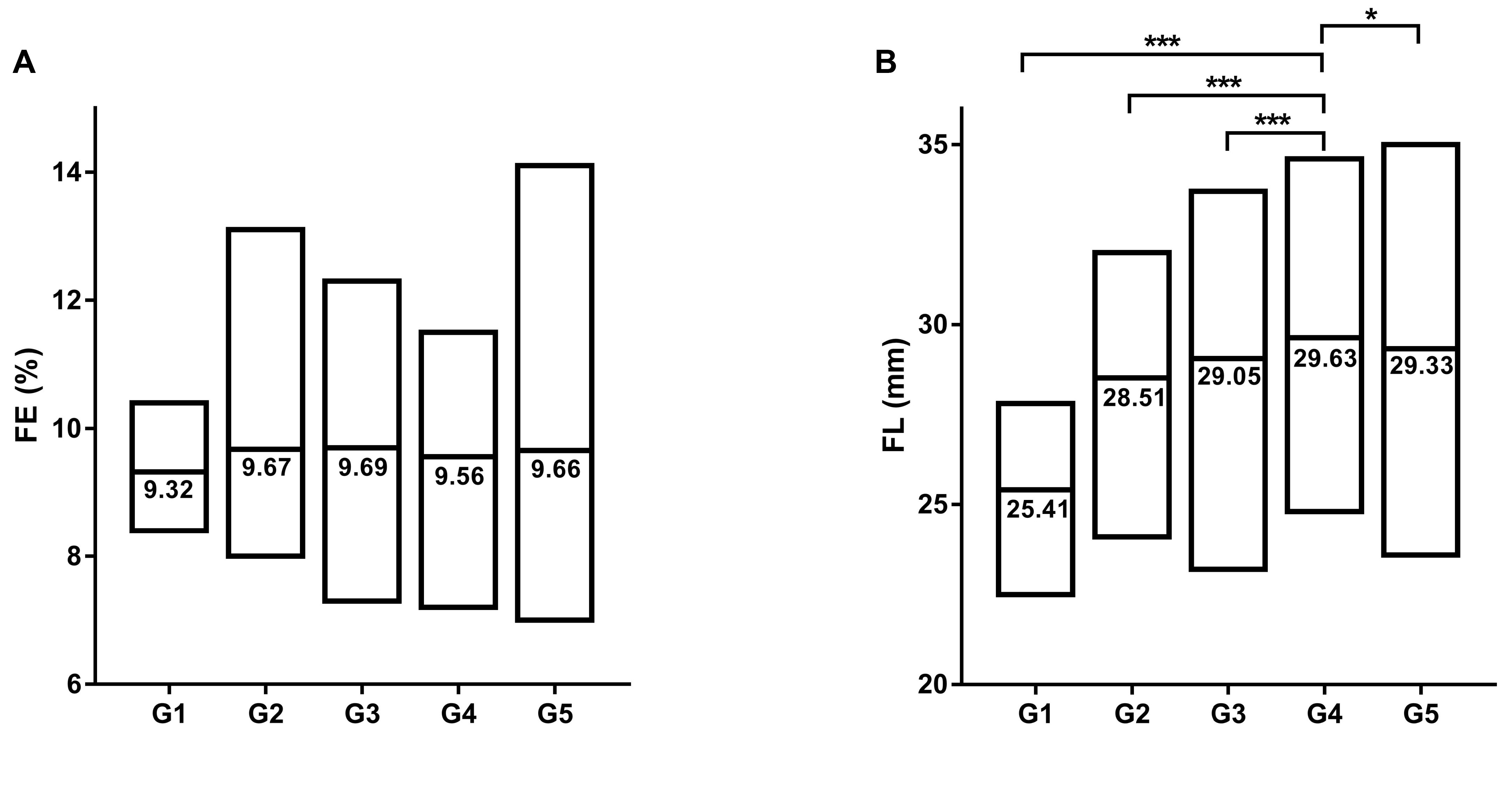

Supplement: Supplementary file 1 [file Image_1.jpeg]
